# The Impact of a Structured Exercise Programme upon Cognitive Function in Chronic Fatigue Syndrome Patients

**DOI:** 10.3390/brainsci10010004

**Published:** 2019-12-19

**Authors:** Paweł Zalewski, Sławomir Kujawski, Malwina Tudorowska, Karl Morten, Małgorzata Tafil-Klawe, Jacek J. Klawe, James Strong, Fernando Estévez-López, Modra Murovska, Julia L. Newton, the European Network on ME/CFS (EUROMENE)

**Affiliations:** 1 Department of Hygiene, Epidemiology and Ergonomics, Collegium Medicum in Bydgoszcz, Nicolaus Copernicus University in Toruń, Poland, M. Sklodowskiej-Curie 9, 85-094 Bydgoszcz, Poland; 2 Gizińscy Medical Centre, Kościuszki 16, 85-079 Bydgoszcz, Poland; 3 Nuffield Department of Women’s & Reproductive Health, The Women Centre, University of Oxford, Oxford OX3 9DU, UK; 4 Department of Human Physiology, Collegium Medicum in Bydgoszcz, Nicolaus Copernicus University in Toruń, Poland, M. Sklodowskiej-Curie 9, 85-092 Bydgoszcz, Poland; 5 Department of Child and Adolescent Psychiatry/Psychology, Erasmus MC University Medical Center, P.O. Box 2040 Rotterdam, The Netherlands; 6 Institute of Microbiology and Virology, Riga Stradiņš University, LV-1067 Riga, Latvia; 7 Institute of Cellular Medicine, The Medical School, Newcastle University, Framlington Place, Newcastle-upon-Tyne NE2 4HH, UK

**Keywords:** mental function, brain fog, cognitive impairment

## Abstract

Background: Cognitive function disturbance is a frequently described symptom of myalgic encephalomyelitis/chronic fatigue syndrome (ME/CFS). In this study, the effects of a structured exercise programme (SEP) upon cognitive function in ME/CFS patients was examined. Methods: Out of the 53 ME/CFS patients initiating SEP 34 (64%) completed the 16 week programme. Cognitive function was assessed using a computerized battery test consisting of a Simple Reaction Time (SRT) (repeated three times) and Choice Reaction Time (CRT) measurements, a Visual Attention Test (VAT) and a Delayed Matching to Sample (DMS) assessment. Results: Statistically significant improvement was noted in the third attempt to SRT in reaction time for correct answers, *p* = 0.045, r = 0.24. Moreover, significant improvement was noted in VAT reaction time, number of correct answers and errors committed, *p* = 0.02, omega = 0.03, *p* = 0.007, r = 0.34 and *p* = 0.004, r = 0.35, respectively. Non-significant changes were noted in other cognitive tests. Conclusions: A substantial number of participants were unwilling or unable to complete the exercise programme. ME/CFS patients able to complete the SEP showed improved visual attention both in terms of reaction time and correctness of responses and processing speed of simple visual stimuli.

## 1. Introduction

Myalgic encephalomyelitis/chronic fatigue syndrome (ME/CFS) is a complex disorder characterised by symptoms including chronic fatigue, exercise intolerance, disordered cognitive functions, autonomic dysfunction, pain, and non–restorative sleep. To date, the specific aetiology of ME/CFS has not been determined [1,2].

The Institute of Medicine (IOM) report in 2015 highlighted an increasing body of literature indicating neurocognitive impairment to be a key symptom in many people with ME/CFS [3]. Cognitive dysfunction includes problems with short-term memory, concentration, attention, processing function, with an impact on everyday activities including work, leisure activities, reducing perceived quality of life [3]. Between 50% to 85% of ME/CFS patients note subjective cognitive impairments [4]. So called “brain fog” is often described by those affected as one of the most debilitating symptoms of the condition [5,6,7,8]. Other aspects of brain function appear unaffected, with higher-level cognitive areas, such as verbal memory, visuo-spatial memory and linguistic fluency appearing normal [9]. However, objective methods of measuring cognitive functioning in those with ME/CFS indicate more global, nonspecific deficits. These primarily include a general slowdown in response speed to tasks that require simple and complex information processing as well as the ability to focus on a task [10]. Studies also confirm that cognitive disturbances of ME/CFS patients are restricted to a decrease in basic processing speed and not related to depression severity [9,11]. Moreover, reduced attention capacity manifesting in disturbed performance on effortful tasks based on planned and self-ordered responses requires the use of short-term memory capacity [12]. Research appears to be suggesting that in ME/CFS some cognitive function domains are more impaired than others with further research needed to quantitatively measure cognitive impairment in ME/CFS and effectiveness of potential therapies

Measuring the efficiency of cognitive function is a research challenge. Although there are theoretical distinctions in the field of cognitive science for brain regions associated with memory, attention and psychomotor performance, in practice there are no tools to measure the individual cognitive functions that usually occur together. Therefore, when the result of a specific neuropsychological test is interpreted as a deficit (e.g., impaired short-memory performance), it is likely that other functions will also be affected as tests usually measure many aspects of cognitive functioning simultaneously [3]. In ME/CFS the mechanisms underlying both subjective and objective cognitive dysfunctions have not yet been thoroughly investigated. Further research is needed to explain the likely biological basis of cognitive deficits [13]. Currently, therapy for those with ME/CFS remains supportive with a focus upon activity management [14]. A structured exercise programme (SEP) for chronic fatigue syndrome has been shown to be effective in some patients, although this remains controversial [15]. Various randomized controlled trials have demonstrated that a form of structured exercise programme for ME/CFS can lead to a significant decrease in fatigue and disabilities [16,17,18]. However, crucially the long-term efficacy of this has been disputed with such participants demonstrating a statistically non-significant difference in fatigue and disability compared to patients allocated to receiving standard medical care (SMC) for at least 2 years follow-up [19,20,21].

There is growing evidence of a link between cardiovascular and muscular deconditioning and cognitive dysfunction in ME/CFS. In a cohort of females with ME/CFS, lower peak heart rate and peak oxygen uptake (assessed during a cycle ergometer exercise task) were associated with a slowed psychomotor response speed [22]. Another study identified a correlation between upper limb muscle function recovery after a fatiguing physical task with information processing speed and sustained attention [23]. These findings suggest that better physical health may lead to improved cognitive outcomes in ME/CFS patients. In fact, improved physical fitness can have positive effects on cognitive functioning [24]. Participants executed selective and persistent attention, cognitive inhibition, and working memory capacity tests. After one week, participants executed fatigue-inducing upper limb exercise. It was found that the return of upper limb muscle function is an important predictor of cognitive performance in patients with CFS [24]. Moreover, significant heterogeneity of examined patients was underlined [24].

Aerobic physical exercise programs have been proposed as an intervention that could improve cognitive function in older people [25] and children [26]. Cvejic et al. [16] examined the effects of a SEP on subjective and objective neurocognitive performance in ME/CFS for the first time. However, more work in this field is needed, therefore the aim of this study was to test the influence of SEP upon functioning of ME/CFS patients with a specific focus in cognitive function. Not all ME/CFS patients can complete a SEP programme and for some it is too difficult. Therefore, in this study we only examine the impact of SEP on ME/CFS patients who managed to complete the 16 week programme.

## 2. Materials and Methods

### 2.1. Study Group

**Figure 1:**
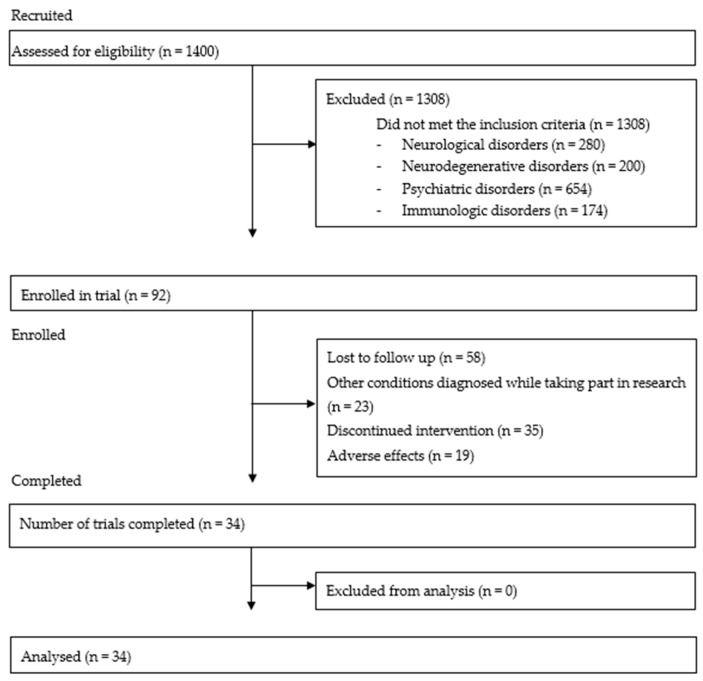
CONSORT-type flow diagram.

Study was conducted in Department of Hygiene, Epidemiology and Ergonomic, Collegium Medicum in Bydgoszcz, Nicolaus Copernicus University in Toruń, Poland. 1400 patients applied to take part in the study and were assessed for eligibility. 1308 were excluded during initial screening due to the presence of other conditions presumably explaining persistent fatigue. Neurological, neurodegenerative, psychiatric and immunologic disorders which were excluding factors comprised those of which mechanisms might presumably explain primary symptoms of ME/CFS. Ninety two volunteers were enrolled into the trial (Figure 1)—all of them were initially diagnosed as CFS patients based on Fukuda criteria [27]. The study was approved by the Ethics Committee, Ludwik Rydygier Memorial Collegium Medicum in Bydgoszcz, Nicolaus Copernicus University, Torun. The 92 patients underwent a baseline assessment that included, body composition analysis, fatigue assessment and autonomic nervous system functioning. Baseline results allowed us to exclude a further 23 patients with other conditions which could explain the presence of persistent fatigue.

Sixty nine patients were invited to attend a second day of assessment which consisted of cardiopulmonary exercise testing (CPET) and explanation of the SEP protocol. However, 16 described themselves as unable to undergo CPET because of the anticipated post-exertional malaise symptoms during and after exertion. In total, 53 patients underwent a SEP protocol of 16 weeks. Thirty four 64.2% patients completed the intervention and underwent a follow up assessment that included fatigue, CPET and autonomic nervous system assessments (Figure 1).

### 2.2. Initial Examination of Patients

#### 2.2.1. Cognitive Function Measurement

To measure cognitive function the computerized battery test—Test Sprawności Operacyjnej (TSO) (software version 4.6.0.44744, Speednet sp. z. o. o., more information available on: http://www.biostat.com.pl/news/nowa_aplikacja_tso_stat_-181.php) was used [28]. The following tests were included: Simple Reaction Time (SRT), Choice Reaction Time (CRT), Visual Attention Test (visual version of match to sample—VAT) and Delayed Matching to Sample (DMS). SRT measures visual information processing speed, CRT is decision making test, VAT measures visual sustained attention, DMS is a test of visual form of short-term memory test. Before starting battery test, short practice of each test was introduced for every patient. Too fast, too slow, or inadequate (wrong or double-pressed key) responses were treated as an error in this battery. At the beginning of every test text instruction was displayed until participant confirm that he/she read it fully by pressing a “space” key on the keyboard. The type of the stimuli was randomly picked from one of five sets: geometrical shapes, plants and animal shapes, arrows, letters or numbers. Proper and distractor stimuli were randomly selected by the software before each trial, all were presented on white background on 15,6”, screen. Whole battery test consisted of subtests in the following order: SRT, CRT, SRT, VAT, DMS and SRT. The SRT test was repeated three times during the test period. On average test duration was 12 min duration (i.e., between the start of whole battery test, and start of the last SRT test). Overall there was a 2 min 20 s interval between start time of first and second SRT test. The following SRT attempts are denoted as SRT.1, SRT.2 and SRT.3.

#### 2.2.2. Structured Exercise Programme

Our SEP protocol is based on the deconditioning and exercise intolerance theories of chronic fatigue syndrome, which could be described as vicious cycle [29]. ME/CFS has been hypothesized to be linked to deconditioning and avoidance of physical activity [29]. Gradual deconditioning eventually leads to an increased effort sensation which leads to an even lower level of physical activity. The aim of SEP is to break this vicious cycle and gradually return the subject to the appropriate level of physical activity level reversing the deconditioning and eventually reducing fatigue and disability [30]. Our SEP is similar to the NICE recommended [14] graded exercise therapy, although initial %HRmax ranged 30–40%, depending on the CPET result. Patients had a period for the first 3–4 weeks adaptation at very low HRmax levels. These modifications have been developed after feedback from patients in our clinic with the aim of improving participation. However, a growing consensus mandating the presence of post exertional malaise (PEM) [3]; a phenomenon which is not apparent in deconditioned healthy controls, in the diagnosis of ME/CFS puts the causative notion of cardiopulmonary deconditioning as principal driver of symptomatology into question. In comparison, Fulcher and White provided programme with initial bouts lasting 15 min at an intensity of 40% of peak oxygen consumption at least 5 days a week [31], while Moss-Morris et al. [24] provided programme with initial bouts duration of 10–15 min 4 to 5 times a week with the intensity of 40 per cent of VO2max. Eventually %HRmax reached 70–80% for 40 min with additional 7 stretching exercises in a training bout in the SEP programme provided in the above study. Fulcher et al. eventually increase the duration to 30 min with a maximum of 60% of peak oxygen consumption while in the case of Moss-Morris target HRmax was 80% for 30 min 5 days a week.

Based on individual CPET results, physiotherapists introduced the patient to a personalised exercise protocol including a demonstration of stretching exercises to be carried out as part of the plan. Home exercise was prescribed at least five days a week, 16 weeks in total, with the initial 3 sessions lasting approximately 10 min. Initially patients were asked to perform training bouts with the intensity of 30–40% of HRmax prescribed individually to patients depending on the CPET result. During the training plan exercise intensity was gradually increased. After the first 3 training sessions, aerobic exercise duration was increased to 20 min with 10% higher intensity in terms of %HRmax; moreover, 3 stretching exercises were added to each training session. Intensity, duration and number of stretching exercises were gradually increased with subjects exercising to between 70–80% of HR max for 40 min with an additional 7 stretching exercises as it was used in previous research [31]. Patients did not exceed their HR max. Telephone calls were made weekly to ensure patients were satisfied with the protocol and identify any problems with compliance. Patients were equipped with heart rate monitors (Beurer PM 25) to help them in sustaining the recommended heart rate. The main exercise was walking however subjects had an opportunity to use other modes of exercise, such as cycling and swimming on request.

#### 2.2.3. Statistical Methods

Shapiro-Wilk test was used to test the assumption of normality. Variables where values did not meet the assumption of distribution normality were analysed using Wilcoxon signed-rank test to compare before vs after intervention. Both T, Z and p values derived from the output of this test are provided. For normally distributed variables repeated-measures ANOVA was used. Effect size for Wilcoxon signed-rank test was calculated based on the following formula [32]:

$$r=\;\frac{Z}{\sqrt{N}}$$

where *Z* is the z-score from applied nonparametric test and n is the size of the study. For repeated-measures ANOVA test, following effect size formula was used [32]:

$$\omega^{2}\;=\frac{\;\left[\frac{k-1}{nk}\left(MS_{M}-MS_{R}\right)\right]}{MS_{R}+\frac{MS_{B}-\;MS_{R}\;}{k}+\left[\frac{k-1}{nk}\left(MS_{M}-MS_{R}\right)\right]}$$

Violin graphs were created using an *R* statistical package [33] with a ggstatsplot library [34]. A Benjamini-Hochberg Adjusted *p* value was chosen to control for False Discovery Rate (FDR) using an online calculator available at (https://tools.carbocation.com/FDR). *p* values both before and after FDR correction are reported.

## 3. Results

### Influence of SEP on Cognitive Function

**Figure 2:**
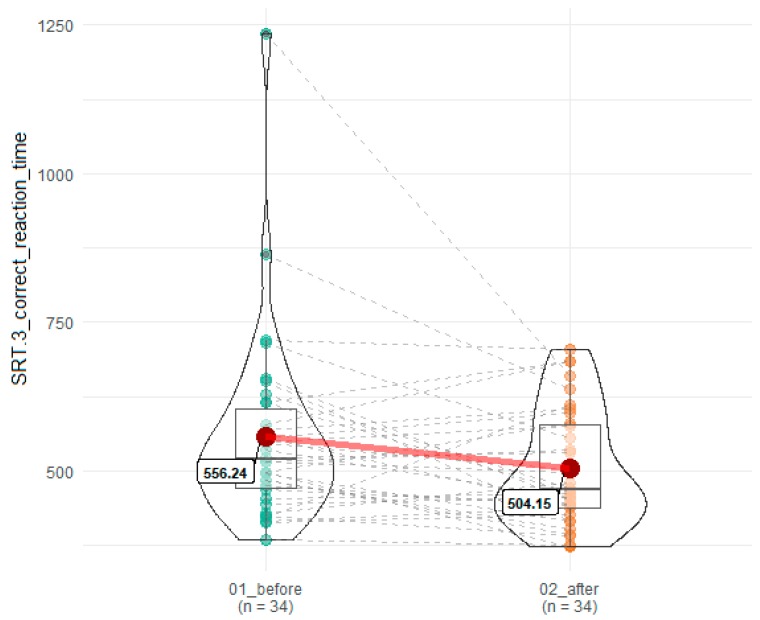
Influence of SEP on reaction time on correct responses in SRT.3. 01_before denotes time point before SEP, while 02_after indicates time point after SEP. Red dots connected by red line indicates mean value, horizontal black line inside the box denotes median value. Green dots before and orange dots after connected by dashed lines denotes results of individual patients. Shape of violin graph indicates distribution of results.

A statistically significant improvement was noted in reaction time for correct answers in the third SRT attempt (SRT3) (556.24 ms in SRT3 before SEP vs 504.15 in SRT3 after), T = 179.5, *z* = 2.02, *p* = 0.045, r = 0.24 (Figure 2).

**Figure 3:**
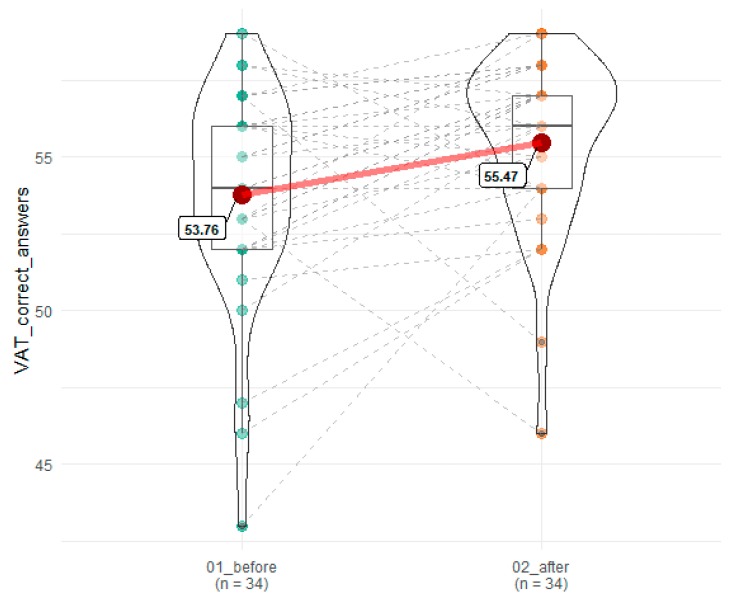
Effects of SEP on number of correct responses in VAT. 01_before denotes time point before SEP, while 02_after indicates time point after SEP. Red dots connected by red line indicates mean value, horizontal black line inside the box denotes median value. Green dots before and orange dots after connected by dashed lines denotes results of individual patients. Shape of violin graph indicates distribution of results.

**Figure 4:**
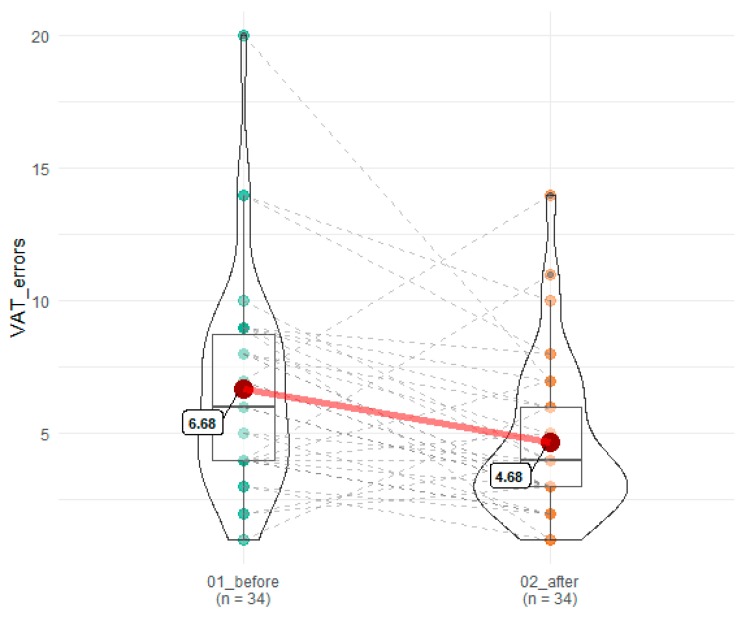
Effects of SEP on errors committed in VAT. 01_before denotes time point before SEP, while 02_after indicates time point after SEP. Red dots connected by red line indicates mean value, horizontal black line inside the box denotes median value. Green dots before and orange dots after connected by dashed lines denotes results of individual patients. Shape of violin graph indicates distribution of results.

A statistically significant improvement was observed in the number of correct answers and errors committed in VAT (53.77 before vs. 55.47 after SEP), T = 118.5, *z* = 2.72, *p* = 0.007, r = 0.34 and 6.68 before vs. 4.68 after SEP), T = 120.5, *z* = 2.86, *p* = 0.004, r = 0.35, respectively (Figure 3 and Figure 4).

**Figure 5:**
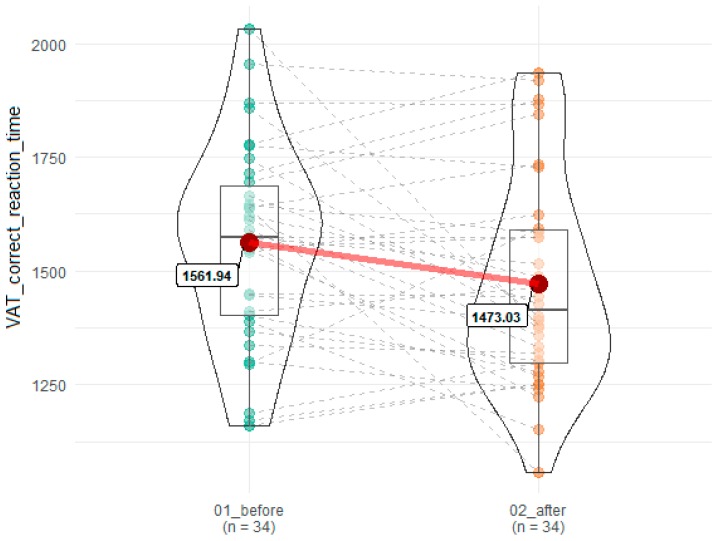
Effects of SEP on reaction time in correct response in VAT. 01_before denotes time point before SEP, while 02_after indicates time point after SEP. Red dots connected by red line indicates mean value, horizontal black line inside the box denotes median value. Green dots before and orange dots after connected by dashed lines denotes results of individual patients. Shape of violin graph indicates distribution of results.

In addition, the reaction time for correct answers in VAT was significantly reduced after SEP (1561.94 ms before vs 1473.03 after, F = 5.78, *p* = 0.02, omega = 0.03) (Figure 5).

No significant changes were observed for other cognitive tests (Table 1). Moreover, none of the statistically significant results survived after FDR correction (Table 1).

## 4. Discussion

The main conclusion of our study is that ME/CFS patients who completed a structured exercise programme improved in terms of (i) correctness of responses in visual attention test, (ii) reaction time in visual attention test, (iii) processing speed of simple visual stimuli. However, a number of patients did not improve and none of the effects remained significant after FDR correction.

Robinson et al. [11] in an examination of cognitive function in ME/CFS patients identified that cognitive disturbances are limited to decrease in basic processing speed. Based on our findings it can be concluded that these aspects of cognitive functioning could be improved in some ME/CFS patients by SEP. Similar results have been obtained in older subjects following physical exercise programmes [25]. In the case of the effects of a physical exercise programme on cognitive function in children, adolescents and young adults Verburgh et al. [25] concluded that more studies are needed, however, they suggested that acute physical exercise improved executive functioning. In a study by Blackwood et al. [35] physical exercise training significantly reduced the decline of ME/CFS patients in a Telephone search task over time, while no significant changes were observed in a Digit Span, Digit Span Backward, Digit Symbol Substitution Test and Word fluency when compared to major depressive disorder patients and healthy controls. In depressive disorders physical exercise programs are an effective non-pharmacological adjunctive therapy [19,36]. Importantly, in this study a diagnosis of psychiatric disease resulted in exclusion of patients from the study. It is important to acknowledge the significant dropout rate (35 from 69 patients (50.7%)) who following CPET assessment or proceeding initiation of the SEP programme did not complete the programme. This is consistent with other studies where a 50% drop out rate was observed within 6–12 months of starting to exercise regularly [37,38,39]. Overall, it could be concluded that both acute physical exercise bout and effects of physical exercise programme can lead to cognitive function improvement in many patient populations. However, in the case of ME/CFS patient’s further research on this topic is needed because participation in a physical exercise programme has the potential to induce post-exertional malaise at least in some participants [40]. The high drop-out rate amongst ME/CFS patients in this study with a number of individuals not showing improved cognitive function highlights the need for care in advocating SEP therapy and the potential heterogeneity of this patient group. It is therefore important, that those who are engaging in a structured exercise programme where there might be potential benefits are fully informed of possible detrimental effects with regular contact with the clinical team. Also research focussing upon risk stratification that allows specific phenotypes who are more likely to benefit from exercise based interventions (and those who are not) is much needed.

Being able to identify sub-groups of patients who benefit or are harmed by any treatment programme before participation is of great importance as we look to find interventions which can improve the lives of ME/CFS patients. At the moment the utmost care must be taken by clinicians in guiding ME/CFS patients exhibiting a worsening of symptomatology following exertion (PEM) to commence a graded exercise programme. In the current study we noted a significant improvement in objective neurocognitive performance in patients with ME/CFS after SEP programme. This is an encouraging observation as it is widely acknowledged that subjective complaints and objective performance indices do not correlate well [17]. Many improvements presented in previous studies are based exclusively on self-report measures [17,41]. Cvejic et al. [16] provide the first evidence of improvements in subjective and objective neurocognitive performance after the completion of a 12-week structured exercise programme incorporating a cognitive training component with objective outcome measures. Also, Cvejic et al. noted an objective neurocognitive performance improvements were accompanied by a significant reduction in responsiveness in stress-related neural pathways consequent to an exercise programme. Further studies could aim to examine the effects of combination of two therapeutic strategies (SEP and self-help neurocognitive training) to maximize improvement after intervention for a select group of ME/CFS patients that do not respond adversely (absence of or lesser severity of PEM) to physical (SEP) and neurocognitive exertion. Moreover, the maintenance of cognitive function improvement with SEP over greater longitude has yet to be determined.

These findings and our results support the idea of the effectiveness of such activity therapies in remediating clinical cognitive status in ME/CFS patients. Our findings warrant further investigation, including replication in a larger sample with proper control group applied but with caveat in that we need to identify potential responders and non-responders before embarking on such programmes.

## Figures and Tables

**Table 1:** Effects of SEP on cognitive function test results.

Variable	Mean (SD) Before	Mean (SD) After	*p*	FDR
SRT.1 correct answers	19.47 (0.99)	19.47 (0.83)	0.89	1.07
SRT.1 errors	0.53 (0.99)	0.53 (0.83)	0.89	1
SRT.1 correct reaction time	547.09 (162.59)	520.56 (129.67)	0.22	0.79
SRT.2 correct answers	19.18 (2.59)	19.71 (0.68)	0.34	1.02
SRT.2 errors	0.82 (2.59)	0.32 (0.68)	0.44	0.88
SRT.2 correct reaction time	540.24 (188.58)	517.32 (106.68)	0.62	0.80
SRT.3 correct answers	19.62 (0.60)	19.59 (0.66)	1.00	1.06
SRT.3 errors	0.38 (0.60)	1.59 (7.33)	1.00	1
SRT.3 correct reaction time	556.24 (157.99)	504.15 (97.28)	0.04	0.18
CRT correct answers	29.24 (0.89)	29.44 (0.75)	0.41	1.05
CRT errors	0.77 (0.89)	0.62 (0.82)	0.53	0.80
CRT correct reaction time	617.97 (175.24)	596.21 (119.01)	0.56	0.78
VAT correct answers	53.77 (3.49)	55.47 (2.85)	0.007	0.06
VAT errors	6.68 (3.88)	4.68 (2.96)	0.004	0.07
VAT correct reaction time	1561.94 (216.66)	1473.03 (232.90)	0.02	0.12
DMS correct answers	22.35 (3.54)	22.94 (3.48)	0.48	0.79
DMS errors	10.82 (3.73)	10.71 (5.58)	0.43	0.97
DMS correct reaction time	1473.59 (265.46)	1418.79 (360.73)	0.47	0.85

## References

[1] Prins J.B., Bleijenberg G., van der Meer J.W.M. (2006). Chronic fatigue syndrome–Authors’ reply. Lancet.

[2] Jason L.A., McManimen S., Sunnquist M., Brown A., Newton J.L., Strand E.B. (2015). Examining the Institute of Medicine’s Recommendations Regarding Chronic Fatigue Syndrome: Clinical Versus Research Criteria. J. Neurol. Psychol..

[3] Institute of Medicine (US) (2015). Committee on the Diagnostic Criteria for Myalgic Encephalomyelitis/Chronic Fatigue Syndrome. Beyond Myalgic Encephalomyelitis/Chronic Fatigue Syndrome: Redefining an Illness.

[4] Logsdon R.G., Gibbons L.E., McCurry S.M., Teri L. (2002). Assessing quality of life in older adults with cognitive impairment. Psychosom. Med..

[5] Komaroff A.L. (1993). Clinical presentation of chronic fatigue syndrome. Chronic Fatigue Syndrome.

[6] Jain S.S., DeLisa J.A. (1998). Chronic fatigue syndrome: A literature review from a physiatric perspective. Am. J. Phys. Med. Rehabil..

[7] Natelson B.H., Lange G. (2002). A status report on chronic fatigue syndrome. Environ. Health Perspect..

[8] Afari N., Buchwald D. (2003). Chronic fatigue syndrome: A review. Am. J. Psychiatry.

[9] Cockshell S.J., Mathias J.L. (2013). Cognitive deficits in chronic fatigue syndrome and their relationship to psychological status, symptomatology, and everyday functioning. Neuropsychology.

[10] Jorgensen R. (2008). Chronic fatigue: An evolutionary concept analysis. J. Adv. Nurs..

[11] Robinson L.J., Gallagher P., Watson S., Pearce R., Finkelmeyer A., Maclachlan L., Newton J.L. (2019). Impairments in cognitive performance in chronic fatigue syndrome are common, not related to co-morbid depression but do associate with autonomic dysfunction. PLoS ONE.

[12] DeLuca J., Johnson S.K., Ellis S.P., Natelson B.H. (1997). Cognitive functioning is impaired in patients with chronic fatigue syndrome devoid of psychiatric disease. J. Neurol. Neurosurg. Psychiatry.

[13] Joyce E., Blumenthal S., Wessely S. (1996). Memory, attention, and executive function in chronic fatigue syndrome. J. Neurol. Neurosurg. Psychiatry.

[14] Baker R., Shaw E.J. (2007). Diagnosis and management of chronic fatigue syndrome or myalgic encephalomyelitis (or encephalopathy): Summary of NICE guidance. BMJ.

[15] Vink M. (2017). PACE trial authors continue to ignore their own null effect. J. Health Psychol..

[16] Cvejic E., Lloyd A.R., Vollmer-Conna U. (2016). Neurocognitive improvements after best-practice intervention for chronic fatigue syndrome: Preliminary evidence of divergence between objective indices and subjective perceptions. Compr. Psychiatry.

[17] Castell B.D., Kazantzis N., Moss-Morris R.E. (2011). Cognitive behavioral therapy and graded exercise for chronic fatigue syndrome: A meta-analysis. Clin. Psychol. Sci. Pract..

[18] Larun L., Brurberg K.G., Odgaard-Jensen J., Price J.R. (2016). Exercise therapy for chronic fatigue syndrome. Cochrane Database Syst. Rev..

[19] Knapen J., Vancampfort D., Moriën Y., Marchal Y. (2015). Exercise therapy improves both mental and physical health in patients with major depression. Disabil. Rehabil..

[20] Wilshire C.E., Kindlon T., Courtney R., Matthees A., Tuller D., Geraghty K., Levin B. (2018). Rethinking the treatment of chronic fatigue syndrome—A reanalysis and evaluation of findings from a recent major trial of graded exercise and CBT. BMC Psychol..

[21] McPhee G. (2017). Cognitive behaviour therapy and objective assessments in chronic fatigue syndrome. J. Health Psychol..

[22] Price J.R., Mitchell E., Tidy E., Hunot V. (2008). Cognitive behaviour therapy for chronic fatigue syndrome in adults. Cochrane Database Syst. Rev..

[23] Ickmans K., Meeus M., Kos D., Clarys P., Meersdom G., Lambrecht L., Nijs J. (2013). Cognitive performance is of clinical importance, but is unrelated to pain severity in women with chronic fatigue syndrome. Clin. Rheumatol..

[24] Moss-Morris R., Sharon C., Tobin R., Baldi J.C. (2005). A randomized controlled graded exercise trial for chronic fatigue syndrome: Outcomes and mechanisms of change. J. Health Psychol..

[25] Verburgh L., Königs M., Scherder E.J., Oosterlaan J. (2014). Physical exercise and executive functions in preadolescent children, adolescents and young adults: A meta-analysis. Br. J. Sports Med..

[26] de Greeff J.W., Bosker R.J., Oosterlaan J., Visscher C., Hartman E. (2018). Effects of physical activity on executive functions, attention and academic performance in preadolescent children: A meta-analysis. J. Sci. Med. Sport.

[27] Fukuda K., Straus S.E., Hickie I., Sharpe M.C., Dobbins J.G., Komaroff A. (1994). The chronic fatigue syndrome: A comprehensive approach to its definition and study. Ann. Intern. Med..

[28] Kujawski S., Słomko J., Tafil-Klawe M., Zawadka-Kunikowska M., Szrajda J., Newton J.L., Zalewski P., Klawe J.J. (2018). The impact of total sleep deprivation upon cognitive functioning in firefighters. Neuropsychiatr. Dis. Treat..

[29] Clark L.V., White P.D. (2005). The role of deconditioning and therapeutic exercise in chronic fatigue syndrome (CFS). J. Ment. Health.

[30] Bavinton J. (2011). Comparison of adaptive pacing therapy, cognitive behaviour therapy, graded exercise therapy, and specialist medical care for chronic fatigue syndrome (PACE): A randomised trial. Lancet.

[31] Fulcher K.Y., White P.D. (1997). Randomised controlled trial of graded exercise in patients with the chronic fatigue syndrome. BMJ.

[32] Field A. (2013). Discovering Statistics Using IBM SPSS Statistics.

[33] R Core Team (2013). R: A Language and Environment for Statistical Computing.

[34] Patil I., Powell C. (2018). Ggstatsplot: ‘ggplot2’ Based Plots with Statistical Details. https://CRAN.R-project.org/package=ggstatsplot.

[35] Blackwood S.K., MacHale S.M., Power M.J., Goodwin G.M., Lawrie S.M. (1998). Effects of exercise on cognitive and motor function in chronic fatigue syndrome and depression. J. Neurol. Neurosurg. Psychiatry.

[36] Hallgren M., Herring M.P., Owen N., Dunstan D., Ekblom Ö., Helgadottir B., Nakitanda O.A., Forsell Y. (2016). Exercise, physical activity, and sedentary behavior in the treatment of depression: Broadening the scientific perspectives and clinical opportunities. Front. Psychiatry.

[37] Annesi J. (2016). Effects of a cognitive behavioral treatment package on exercise attendance and drop out in fitness centers. Eur. J. Sport Sci..

[38] Annesi J.J. (1998). Effects of computer feedback on adherence to exercise. Percept. Mot. Ski..

[39] Marcus B.H., Forsyth L.H., Stone E.J., Dubbert P.M., McKenzie T.L., Dunn A.L., Blair S.N. (2000). Physical activity behavior change: Issues in adoption and maintenance. Health Psychol..

[40] Chu L., Valencia I.J., Garvert D.W., Montoya J.G. (2018). Deconstructing post-exertional malaise in myalgic encephalomyelitis/chronic fatigue syndrome: A patient-centered, cross-sectional survey. PLoS ONE.

[41] Deale A., Husain K., Chalder T., Wessely S. (2001). Long-term outcome of cognitive behavior therapy versus relaxation therapy for chronic fatigue syndrome: A 5-year follow-up study. Am. J. Psychiatry.

